# Patient and Care Partner Reactions to a Primary Care Brain Health Visit for Cognitive Assessment

**DOI:** 10.1093/geroni/igaf122.4340

**Published:** 2025-12-31

**Authors:** Clarissa Hsu, Mikael Anne Greenwood-Hickman, Alana Elop, Leah Karliner, Tyler Barrett, Leonardo Coleman, Sascha Dublin, Deborah Barnes

**Affiliations:** Kaiser Permanente Washington, Seattle, Washington, United States; Kaiser Permanente Washington, Seattle, Washington, United States; UCSF, San Francisco, California, United States; UCSF, San Francisco, California, United States; Kaiser Permanente Washington, Seattle, Washington, United States; Kaiser Permanente Washington, Seattle, Washington, United States; Kaiser Permanente Washington, Seattle, Washington, United States; University of California, San Francisco, San Francisco, California, United States

## Abstract

The eRADAR study is a randomized controlled trial in two United States healthcare systems testing targeted dementia screening in primary care. High-risk individuals ≥ age 65 were identified using a predictive algorithm—eRADAR (electronic health record Risk of Alzheimer’s and Dementia Assessment Rule)—and invited for a “brain health” screening visit (BHV). Patients and their primary care providers (PCPs) were given visit findings (possible dementia, possible mild cognitive impairment [MCI], or normal cognition) and follow-up recommendations. A subset of participants and their care partners (CPs) were invited to complete in-depth telephone interviews based on a purposeful sampling that prioritized participants who screened positive for possible dementia or MCI. We conducted interviews representing 40 BHVs (38 patients and 19 CPs). Forty-eight percent of patients screened positive for possible dementia, 38% for MCI, and 15% were cognitively normal. Patient reactions to the BHV were positive overall. Very few participants reported that the BHV caused distress or anxiety, but they also did not feel the BHV provided them with new information. Patients and CPs often downplayed results suggestive of cognitive impairment or attributed them to other causes, and many were reluctant to follow up with their PCP. Interview feedback indicates the BHV did not consistently motivate people to seek further evaluation or care for cognitive issues. Similar screening programs should consider more direct options to motivate follow-up for positive findings (e.g., directly scheduling a follow-up with the PCP, direct referral to specialty care, screening immediately before a PCP appointment).

